# Structural insight into mechanisms for dynamic regulation of PKM2

**DOI:** 10.1007/s13238-015-0132-x

**Published:** 2015-02-04

**Authors:** Ping Wang, Chang Sun, Tingting Zhu, Yanhui Xu

**Affiliations:** 1 Fudan University Shanghai Cancer Center and Institute of Biomedical Sciences, Shanghai Medical College of Fudan University, Shanghai, 200032 China; 2 State Key Laboratory of Genetic Engineering, School of Life Sciences, Fudan University, Shanghai, 200433 China

**Keywords:** pyruvate kinase M2, crystal structure, allosteric regulation, Warburg effect, post-translational modifications

## Abstract

Pyruvate kinase isoform M2 (PKM2) converts phosphoenolpyruvate (PEP) to pyruvate and plays an important role in cancer metabolism. Here, we show that post-translational modifications and a patient-derived mutation regulate pyruvate kinase activity of PKM2 through modulating the conformation of the PKM2 tetramer. We determined crystal structures of human PKM2 mutants and proposed a “seesaw” model to illustrate conformational changes between an inactive T-state and an active R-state tetramers of PKM2. Biochemical and structural analyses demonstrate that PKM2^Y105E^ (phosphorylation mimic of Y105) decreases pyruvate kinase activity by inhibiting FBP (fructose 1,6-bisphosphate)-induced R-state formation, and PKM2^K305Q^ (acetylation mimic of K305) abolishes the activity by hindering tetramer formation. K422R, a patient-derived mutation of PKM2, favors a stable, inactive T-state tetramer because of strong intermolecular interactions. Our study reveals the mechanism for dynamic regulation of PKM2 by post-translational modifications and a patient-derived mutation and provides a structural basis for further investigation of other modifications and mutations of PKM2 yet to be discovered.

**Electronic supplementary material:**

The online version of this article (doi:10.1007/s13238-015-0132-x) contains supplementary material, which is available to authorized users.

## Introduction

Most somatic cells produce energy predominantly through oxidative phosphorylation, whereas cancer cells produce energy mainly through the less efficient glycolytic pathway, followed by lactic acid production under aerobic conditions (Warburg, 1956). This aerobic glycolysis (also known as the Warburg effect) plays an important role in tumorigenesis (Vander Heiden et al., 2009; Koppenol et al., 2011; Chaneton and Gottlieb, 2012; Yang and Lu, 2013; Wong et al., 2015). Pyruvate kinase transfers one phosphate group from phosphoenolpyruvate (PEP) to adenosine di-phosphate (ADP), and thus generates one pyruvate molecule and one adenosine tri-phosphate (ATP) molecule. This step is one of the rate-limiting steps of glycolysis, and therefore pyruvate kinase is one of the primary mediators of glycolysis.

Four mammalian isoforms of pyruvate kinase-PKL, PKR, PKM1 and PKM2-are expressed in various types of cells and tissues. PKL is expressed in the liver, and PKR is expressed in the red blood cells. PKM1 and PKM2 are derived from alternative splicing of the *PKM* gene (Noguchi et al., 1986; Noguchi et al., 1987). PKM1 is a constitutively active isoform expressed in differentiated cells from various tissues. In contrast, PKM2 has low basal activity and is activated by an effector molecule fructose 1,6-bisphosphate (FBP). PKM2 is expressed in most proliferating cells (Christofk et al., 2008a). Furthermore, the regulation of PKM2 pyruvate kinase activity plays an essential role in cancer metabolism and is crucial for the growth and survival of cancer cells (Chaneton and Gottlieb, 2012; Yang and Lu, 2013; Wong et al., 2015). Recently, PKM2 has been reported to function as a nuclear protein kinase to regulate gene transcription and promote tumorigenesis (Gao et al., 2012; Yang et al., 2012a, b; Gao et al., 2013; Lv et al., 2013; Keller et al., 2014).

PKM2 exists in a dynamic population of monomer, dimer and tetramer and its pyruvate kinase activity relies on the formation of the tetramer. Upon formation of the tetramer, PKM2 can adopt the inactive T-state or active R-state conformation (Morgan et al., 2013). The pyruvate kinase activity of PKM2 is regulated by metabolic intermediates and post-translational modifications. For example, metabolic intermediates such as FBP and *N*-succinyl-5-aminoimidazole-4-carboxamide ribose-5′-phosphate (SAICAR) increase the pyruvate kinase activity of PKM2 (Dombrauckas et al., 2005; Chaneton et al., 2012; Keller et al., 2012). Synthetic compounds have been screened for their ability to activate PKM2 and, thus, suppress cancer growth (Boxer et al., 2010; Jiang et al., 2010; Anastasiou et al., 2012; Kung et al., 2012). Some of these compounds have been shown to promote tetramer formation of PKM2 and enhance its enzymatic activity (Anastasiou et al., 2012). In addition, serine and phenylalanine have been reported to bind to the allosteric site of PKM2 and activate or inhibit its activity, respectively (Chaneton et al., 2012; Morgan et al., 2013).

Post-translational modifications including phosphorylation, oxidation, hydroxylation and acetylation also regulate PKM2 activity to promote cancer proliferation. For example, acetylation of residue K305 inhibits pyruvate kinase activity of PKM2 *in* *vitro* (Lv et al., 2011) and acetylation of residue K433 affects FBP binding and prevents PKM2 activation (Lv et al., 2013). Phosphorylation of residue Y105 inhibits the tetramer formation and pyruvate kinase activity of PKM2 (Hitosugi et al., 2009). Furthermore, oxidation of residue C358 inhibits PKM2 activity and promotes the metabolic changes required for proliferation (Anastasiou et al., 2011). PKM2 hydroxylation of P403 and P408 promotes HIF-1 transactivation in cancer cells (Luo et al., 2011). The mutation R399E of PKM2 (PKM2^R399E^) was shown to disrupt the tetramer formation on one of dimer interfaces, thereby producing dimers and decreasing its pyruvate kinase activity (Gao et al., 2012). In addition, mutations K422R and H391Y of PKM2 (PKM2^K422R^ and PKM2^H391Y^) were shown to decrease its pyruvate kinase activity in Bloom Syndrome (BS) patients, who are prone to cancer (Anitha et al., 2004; Akhtar et al., 2009; Gupta et al., 2010; Iqbal et al., 2014).

Although PKM2 has been studied for decades, how its activity is regulated remains poorly understood. In this study, we revealed how pyruvate kinase activity of PKM2 is regulated by post-translational modifications and a patient-derived mutation. On the basis of our observations, we propose a model for dynamic regulation of PKM2. Our study also provides a structural basis for further investigation of dynamic regulation of PKM2 by other post-translational modifications and mutations involved in cancer metabolism.

## Results

### Effects of post-translational modifications and a patient-derived mutation on PKM2 activity

**Figure 1:**
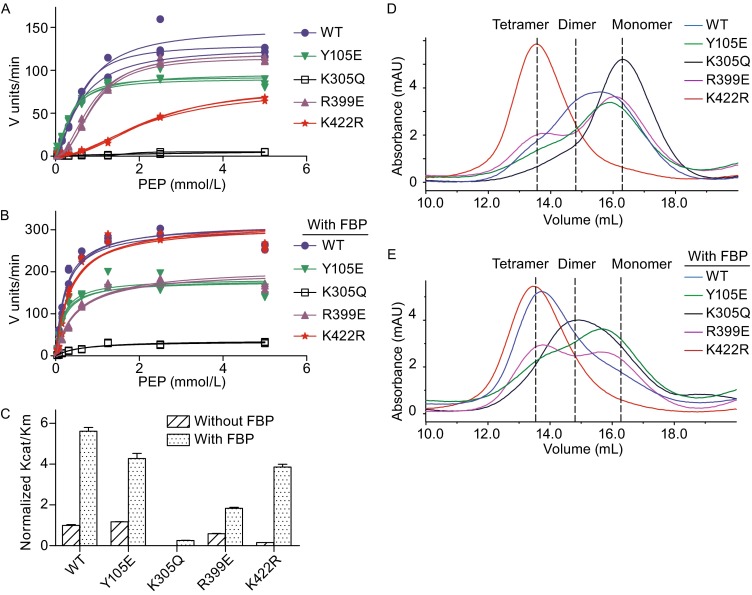
**Activities and tetramer formation for wild-type PKM2 and mutants of PKM2**. (A and B) Kinetic activities of PKM2 proteins in the absence (A) or presence (B) of FBP. The data were fit with the Allosteric Sigmoidal Equation (A) or the Michaelis-Menten Equation (B). (C) Normalized activities (kcat/Km) of wild-type PKM2 and mutants of PKM2 calculated according to the results from Fig. 1A and 1B with the value (kcat/Km) of PKM2^WT^ as a standard. The error bars represent mean ± SD for triplicate experiments. (D and E). Gel filtration of wild-type and mutants of PKM2 in the absence (D) or presence (E) of FBP. Peak positions of monomer, dimer and tetramer are indicated as dashed lines. The color scheme for PKM2 proteins is indicated. Superdex 200 (GE Healthcare, 10/300 GL) was used in gel-filtration analyses

To investigate how the enzymatic activity of PKM2 is regulated, we purified wild-type PKM2 (PKM2^WT^), PKM2^R399E^ (a dimeric mutant) (Gao et al., 2012) and PKM2^K422R^ (a patient-derived mutation). Given the difficulty to obtain phosphorylated or acetylated PKM2 proteins, we purified PKM2^Y105E^ (a phosphorylation mimic of Y105) and PKM2^K305Q^ (an acetylation mimic of K305) to mimic PKM2 containing the two modifications. The above four PKM2 mutants and PKM2^WT^ were used for enzymatic activity assays and structural studies (Fig. S1A). We first measured the enzymatic activities for wild-type and mutants of PKM2 and calculated the Km values for PEP. The kinetic activities were calculated based on the PEP saturation curves in the absence or presence of FBP, an allosteric activator of PKM2 (Fig. 1A and 1B). We also calculated the normalized pyruvate kinase activity (kcat/Km, (mol/L)^−1^ s^−1^), as represented by the value of Vmax/Km of PKM2^WT^, because an equal amount of protein was used for all activity assays (Fig. 1C). We could not detect protein kinase activity for human PKM2 under our experimental conditions using histone H3 as substrate (data not shown). Therefore, only pyruvate kinase activities (enzymatic activity hereafter if not specified) were measured for wild-type and mutants of PKM2. A high enzyme concentration in the reaction would greatly decrease the accuracy of calculated reaction rate. Because of the extremely fast reaction rate (less than 1.5 min), we measured the pyruvate kinase activity in nanomolar concentrations.

In the presence of FBP, PKM2^WT^ showed a significant increase in enzymatic activity (compared to that in the absence of FBP) with a normalized kcat/Km of 5.6 and a decrease in Km to 0.2 mM (Fig. 1A–C and Table S1). PKM2^K305Q^ showed barely detectable pyruvate kinase activity in the absence of FBP, and a normalized kcat/Km of 0.2 in the presence of FBP, indicating a significant decrease in activity. Compared to PKM2^WT^, PKM2^R399E^ showed a decrease in activity in the absence or presence of FBP, with a kcat/Km of 0.6 or 1.8, respectively. Interestingly, PKM2^Y105E^ showed a slight decrease in activity in the presence, but not the absence, of FBP, with a kcat/Km of 1.2 in the absence of FBP and 4.3 in the presence of FBP. In contrast, PKM2^K422R^ significantly decreased the enzymatic activity (kcat/Km of 0.2) and substrate binding affinity only in the absence of FBP. Taken together, these mutations showed different effects on pyruvate kinase activity of PKM2.

### Enzymatic activities of PKM2 mutants are not positively correlated with PKM2 tetramer formation

Tetramer formation is essential for the pyruvate kinase activity of PKM2 (Dombrauckas et al., 2005). The gel-filtration analyses of wild-type and mutants of PKM2 show a mixed population of PKM2 in monomer, dimer and tetramer (Figs. 1D, 1E and S1B). PKM2^WT^ appears to preferentially form a dimer in the absence of FBP and tends to form a tetramer in the presence of FBP. Compared to PKM2^WT^, PKM2^Y105E^ and PKM2^R399E^ showed similar patterns of oligomerization in the absence of FBP. In contrast, PKM2^K305Q^ tended to form a monomer whereas PKM2^K422R^ mainly formed a tetramer in the absence of FBP. In the presence of FBP, PKM2^Y105E^ and PKM2^R399E^ only slightly increased their tendency to form a tetramer, whereas PKM2^K305Q^ only formed a dimer; PKM2^K422R^ maintained its tendency to form the tetramer. The above observations are generally consistent with the prediction that the more tetramer that forms, the more active the protein is. However, exceptions exist. For example, in the absence of FBP, PKM2^K422R^ showed a significantly high level of tetramer formation, but was much less active than PKM2^WT^. The results indicate that tetramer formation alone cannot explain how PKM2 activity is regulated by these mutations.

It was also noted that PKM2^WT^ formed a tetramer in a concentration- and time-dependent manner (Fig. S1C and S1D). As indicated by size exclusive chromatography, the higher concentration of protein used, the more tetramer formed (Fig. S1C). Interestingly, monomeric PKM2 eluted when either tetrameric or monomeric PKM2 was re-subjected to size exclusion chromatography under similar experimental conditions (Fig. S1D). To minimize concentration- and time-dependent confounding factors, we compared the oligomerization states of PKM2^WT^ and all the mutants under similar protein concentrations and experimental conditions.

### Enzymatic activities of PKM2 mutants are not positively correlated with their thermal stability

The thermal stability of PKM2 was reported to reflect its enzymatic activity (Morgan et al., 2010). Thus, we next performed a thermal-shift assay to investigate the correlation between enzymatic activity and thermal stability of PKM2 proteins. PKM2^WT^ and PKM2^Y105E^ showed two peaks (Tm: ~44°C and ~60°C) in the absence of FBP and a single peak (Tm value of ~59°C) in the presence of FBP (Fig. S1E, Table S1). Compared to PKM2^WT^, PKM2^K305Q^ was less stable in the absence (Tm: ~44°C) and presence (Tm: ~54°C) of FBP, which is consistent with its lesser tendency toward tetramer formation and lower enzymatic activity. The Tm values of PKM2^R399E^ (~ 56°C) and PKM2^K422R^ (~ 61°C) were slightly enhanced by the addition of FBP, indicating that both proteins are relatively stable and FBP could not significantly enhance their thermal stabilities. These results indicate that thermal stability is not positively correlated with enzymatic activity or tetramer formation of the wild-type and mutants of PKM2 tested in this study. Taken together, the above results indicate that changes of the pyruvate kinase activity of PKM2 could not be predicted based on the state of oligomerization or thermal stability.

### Crystal structures of human PKM2 mutants

To investigate the molecular mechanism for the regulation of PKM2 activity, we determined the crystal structures of PKM2^K305Q^, PKM2^Y105E^, PKM2^R399E^ and PKM2^K422R^ in the absence of FBP, in addition to PKM2^K422R^ in the presence of FBP (PKM2^K422R_FBP^) (Fig. S2A and Table 1). Although PKM2^WT^ and PKM2 mutants (except PKM2^K422R^) were present in the form of a monomer in a certain percentage in solution with low protein concentration (less than 0.2 mg/mL) (Fig. 1D), all PKM2 proteins formed tetramer in the crystals. The results are consistent with the observation that higher protein concentration leads to more tetramer formation for PKM2 proteins in gel filtration (Fig. S1C). The crystal structures of wild-type PKM2 in the presence of oxalate (PKM2^Oxalate^) (PDB: 3BJT) (Christofk et al., 2008b) and PKM2 in complex with phenylalanine (PKM2^Phe^) (PDB: 4FXJ) (Morgan et al., 2013) were used for the following structural comparison. In addition, we drafted a model for four monomers in the tetramer structure to clarify the following description (Fig. S2A).

**Table 1:** **Crystallographic data and structure refinement statistics**

Protein	PKM2^Y105E^	PKM2^K305Q^	PKM2^R399E^	PKM2^K422R^	PKM2^K422R_FBP^
*Data collection*					
Wavelength (Å)	0.97927	0.97927	0.97923	0.97923	0.97923
Resolution range (Å)	50–3.2 (3.31–3.20)^a^	50–2.3 (2.38–2.30)	50–2.4 (2.49– 2.40)	50–2.3 (2.38–2.30)	50–2.6 (2.69–2.60)
Space group	*P* 31	*P* 1 21 1	*P* 1 21 1	*P* 1 21 1	*P* 1 21 1
Unit cell (Å, °)	124.5, 124.5, 257.0, 90.0, 90.0, 120.0	94.8, 117.4, 110.3, 90.0, 113.2, 90.0	95.6, 71.2, 170.3, 90, 104.3, 90	97.9, 70.9, 169.5, 90.0, 100.2, 90.0	81.7, 152.6, 97.7, 90.0, 104.2, 90.0
Total reflections	610595	610809	513532	581223	411179
Unique reflections	72016	98003	87781	98424	70893
Multiplicity	8.5 (8.3)	6.2 (6.3)	5.8 (5.8)	5.9 (5.9)	5.6 (5.8)
Completeness (%)	98.72 (88.38)	99.52 (95.59)	98.30 (84.27)	96.72 (89.08)	98.9 (100)
Mean *I*/sigma (*I*)	5.56 (2.87)	11.35 (3.33)	7.34 (3.91)	7.47 (2.93)	35.0 (2.68)
*R*-sym	0.192 (0.768)	0.098 (0.552)	0.166 (0.83)	0.137 (0.676)	0.084 (0.854)
*Structure refinement*					
*R*-factor	0.2202	0.2131	0.2101	0.1974	0.2759
*R*-free^b^	0.2652	0.2601	0.2562	0.2397	0.3036
RMS (bonds)	0.010	0.002	0.002	0.002	0.005
RMS (angles)	1.39	0.63	0.57	0.60	0.87
Average B-factor	40.90	25.20	32.30	37.80	89.70
*Ramachandran plot statistics*					
Most favored regions (%)	84.2	91.8	92.2	90.7	93.5
Allowed regions (%)	14.1	7.9	7.2	8.6	5.8
Generously allowed regions (%)	1.4	0.3	0.3	0.4	0.4
Disallowed regions (%)	0.3	0	0.3	0.3	0.3

^a^ The values for the data in the highest resolution shell are shown in parentheses

^b^ *R*free = ∑Test||*F*obs|  − |*F*calc||/∑Test |*F*obs|, where “Test” is a test set of about 5% of the total reflections randomly chosen and set aside prior to refinement for the structure

The first striking observation from the structural analyses is that PKM2^Oxalate^, PKM2^K305Q^ and PKM2^K422R_FBP^ adopted the R-state conformation, whereas all other PKM2 mutants adopted the T-state conformation (Fig. S2A). Notably, phenylalanine was previously reported to function as an inhibitor to lock PKM2 in an inactive T-state conformation (Morgan et al., 2013). PKM2^Oxalate^ adopted an R-state conformation because the crystals were obtained in the presence of oxalate (an analog of PEP), which may induce an R-state conformation of PKM2 (Christofk et al., 2008b). Similarly, PKM2^K305Q^ adopted an R-state conformation because the protein was crystallized in the presence of malonate (0.2 mol/L), which was observed in the active site of the PKM2^K305Q^ structure (Fig. S2B). Both oxalate and malonate are analogs of PEP and may favor the formation of the R-state tetramer. These results suggest that wild-type PKM2 and mutants of the PKM2 tetramer tend to adopt the T-state conformation in the apo form but prefer an R-state conformation in the presence of either FBP or an analog of PEP.

### A “seesaw” model in the R-/T-state transition of PKM2

**Figure 2:**
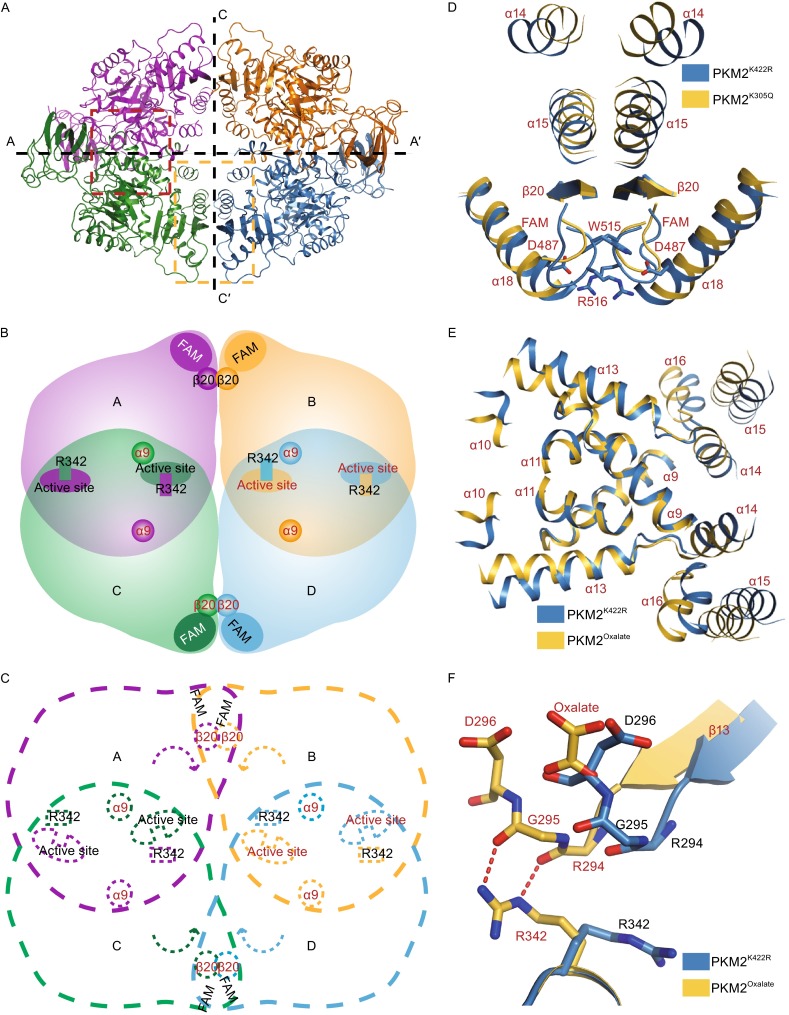
**Crystal structure of human PKM2 and a “seesaw” model for conformational transitions of PKM2 tetramer**. (A) Ribbon representation of the human PKM2 structure (PKM2^K422R^) with A-A′ (red box) and C-C′ (yellow box) interfaces indicated as dashed lines. Four monomers are shown in different colors. (B and C) A “seesaw” model for the conformational transitions between the R-state (B) and T-state (C) conformations of the PKM2 tetramer. Critical elements for the conformational changes are indicated. Dashed arrows indicate the directions for the rotation of each monomer from the R- (B) to the T- (C) state. The monomers are indicated as monomer A to D for simplicity in the following description. (D and E) A close-up view for the structural comparison of the PKM2 structure in the R- and T-state conformations on the C-C′ (D) or A-A′ (E) interface. PKM2 in R- and T-state conformations are colored in yellow and blue, respectively. (F) A structural comparison of the active site of PKM2 in R- and T-state tetramers. PKM2 in R- and T-state conformations are colored in yellow and blue, respectively

Previous studies have demonstrated that a PKM2 tetramer in the R-state (active) is more active than that in the T-state (inactive). Structural comparison indicates that each individual monomer of PKM2 adopts similar fold with a root-mean-squared deviation (rmsd) of less than 0.6 Å for approximately 430 aligned Cα atoms (Fig. S2C). However, structure analysis indicates that the PKM2 tetramer undergoes significant changes between R- and T-state conformations with a rotation about the helix α9 of five degrees for each monomer (Fig. 2B and 2C). This observation is consistent with previous studies of the PKM protein in *Leishmania Mexicana* (Morgan et al., 2010). The PKM2 tetramer is formed through intermolecular interactions between four monomers on large (A-A′) and small (C-C′) interfaces (Fig. 2A). We propose a seesaw model for overall conformational changes during transitions between the R-state (PKM2^K305Q^) and the T-state (PKM2^K422R^) (Fig. 2B and 2C).

On the C-C′ interface, each monomeric PKM2 adopts a rigid conformation and rotates alone with strand β20 (Fig. 2B–D). When PKM2 adopts the T-state conformation, the FBP activation motif (FAM, residues G514–T522) and helix α18 on monomer A interact with FAM and α18 on monomer B on one side of two parallel seesaws. Helices α14/α15 on monomer A and α14/α15 on monomer B are apart from one another on the other side (Figs. 2D and S3). In a process of transitioning from the T-state to the R-state, FAM/α18 on monomer A and FAM/α18 on monomer B move away from one another, and helices α14/α15 on monomer A and α14/α15 on monomer B move closer to one another. Consistent with the above observations, the distance between residues M525 (β20) on monomer A and M525 (β20) on monomer B is approximately 5.0 Å in either the R- or T-state tetramer (Table S2). In contrast, the distance between residues L488 (in α18) on monomer A and L488 (in α18) on monomer B is ~25.9 Å in the T-state and ~27.4 Å in the R-state conformation, respectively; the distance between residues E397 (in α14) on monomer A and E397 (in α14) on monomer B is ~20.7 Å in T-state and ~14.0 Å in the R-state conformation.

On the A-A′ interface, each monomeric PKM2 rotates alone with helix α9 (Fig. 2E). When PKM2 adopts the R-state conformation, oxalate is recognized in the active site by residues R294, G295 and D296 of α8 on monomer A. The three residues are stabilized by residue R342 of α11 on monomer C through forming two intermolecular hydrogen bonds. In the process of an R- to T-state transition, helices α10/α11/α13 on monomer A move away from α10/α11/α13 on monomer C, whereas helices α14/α15/α16 on monomer A move closer to α14/α15/α16 on monomer C. The conformational change leads to a flip of the side chain on residue R342 on monomer C, which stabilizes residues R294/G295/D296 on monomer A in the R-state conformation. As a result, PKM2 forms an inactive T-state tetramer, in which oxalate is not well recognized in the active site (Fig. 2F). The mutation R342 W of PKM2 abolished its enzymatic activity, further supporting its significance for catalysis (Fig. S1G). Consistent with above observations, the distances between residues K311 (in α9) on monomer A and K311 (in α9) on monomer C are approximate 18.0 Å in the two states (Table S2). In contrast, the distance between residues E373 (in α13) on monomer A and E373 (in α13) on monomer C is ~37.8 Å in the T-state and ~31.1 Å in the R-state conformation; the distance between residues V414 (in α15) on monomer A and V414 (in α15) on monomer C is ~36.3 Å in the T-state and ~40.5 Å in the R-state conformation.

In support of the above conformational changes, the transition of PKM2 from the R-state to the T-state, the distance between β20 on monomer A and β20 on monomer C along with the C-C interface decreases from ~59.6 Å to ~53.6 Å, whereas the distance between α9 on monomer A and α9 on monomer B along with the A-A′ interface increases from ~43.4 Å to ~51.6 Å (Table S2). Regulation of intermolecular interactions on either the A-A′ (PEP binding site) or C-C′ (α14/α15 or FAM/α18) interface will lead to an R-/T-state transition of PKM2 and affect its enzymatic activity. For example, association of PEP or its analog oxalate of PKM2 induces an R-state conformation through enhancing intermolecular interactions on the A-A′ interface. In addition, FBP recruits FAM back to the FBP binding pocket of PKM2 and disrupts the intermolecular interactions on the C-C′ interface to form an R-state conformation (Fig. 2B and 2C).

### PKM2^K305Q^ disrupts tetramer formation and impairs enzymatic activity

**Figure 3:**
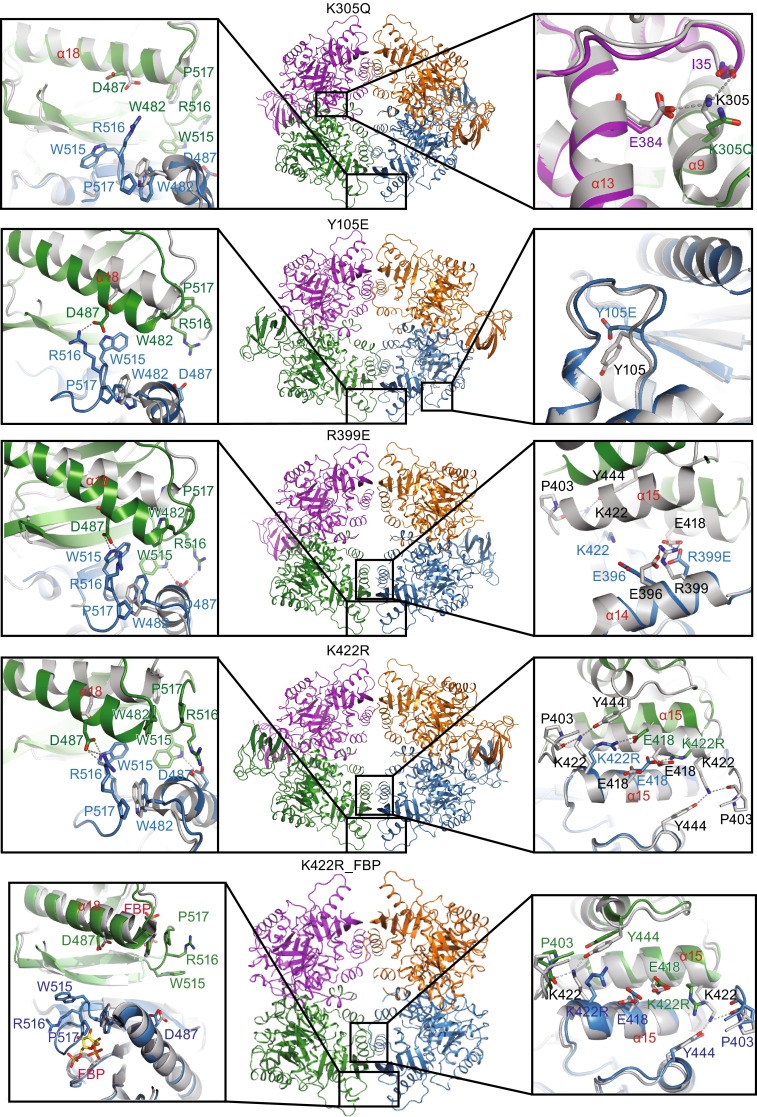
**Structural comparisons of PKM2 mutants and PKM2** ^**Oxalate**^ **(in the R-state)**. Close-up views of PKM2 mutants with critical residues indicated as stick representations. PKM2^Oxalate^ (in the R-state) is colored in gray

PKM2^K305Q^ adopts a similar R-state conformation to that of PKM2^Oxalate^ with a rmsd of 0.48 Å for 1750 aligned Cα atoms (Fig. 3). The existence of malonate in the condition of crystallization may lead to an active R-state conformation of PKM2^K305Q^ (Fig. S2B). No significant conformational change was observed for the two compared structures (Fig. S2A). However, differences exist between PKM2^K305Q^ and PKM2^Oxalate^. In the PKM2^Oxalate^ structure, two hydrogen bonds are formed between (1) residues K305 (on helix α9 of monomer C) and E384 (on helix α13) on monomer A and (2) K305 and I35 (close to helix α1) on monomer A (Fig. 3). The hydrogen bonds facilitate the intermolecular interactions on the A-A′ interface. No such contact was observed in PKM2^K305Q^ structure.

The FBP binding pocket on the C-C′ interface of PKM2^K305Q^ are structurally similar to that of PKM2^Oxalate^. Consistently, the enzymatic activity of PKM2^K305Q^ could be slightly elevated by FBP (Fig. 1C). Gel-filtration and thermal-shift assays demonstrated that FBP retains its ability to stabilize PKM2^K305Q^ and promote its dimer formation (Figs. 1D, 1E and S1E). Altogether, mutation K305Q or acetylation of residue K305 inhibits PKM2 activity through hindering the tetramer formation on A-A′ interface.

### PKM2^K422R^ forms a tetramer in the T-state in the absence of FBP and the R-state in the presence of FBP

Different from PKM2^Oxalate^ or PKM2^K305Q^, PKM2^K422R^ adopts a T-state conformation in the absence of FBP and an R-state conformation in the presence of FBP (Fig. S2A). In the PKM2^Oxalate^ structure, two hydrogen bonds are formed between residue K422 (on monomer C) and residues P403 and Y444 (on monomer D), whereas FAM is flexible and invisible (Fig. 3). In the PKM2^K422R^ structure, residue K422R on monomer C forms two hydrogen bonds with residue E418 on monomer D. Intermolecular interactions are mediated by hydrogen bonds between residue D487 on α18 of monomer C and W515 and R516 on FAM of monomer D, and hydrophobic interactions between residues W482, W515 and P517 on both monomer C and monomer D. The FBP association stabilizes the FAM and disrupts the interactions between FAM on monomer C and α18 on monomer D, therefore allowing the tetramer to adopt an R-state conformation (Fig. 3).

**Figure 4:**
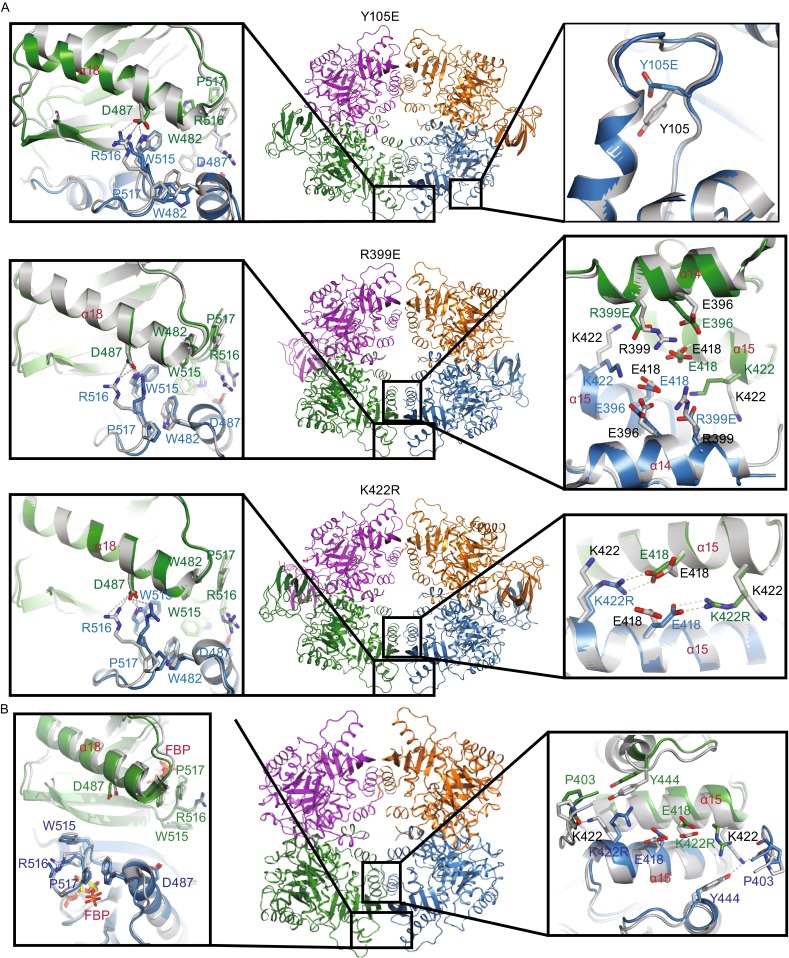
**Structural comparisons of PKM2 mutants with PKM2** ^**Phe**^ **(in T-state) and PKM2** ^**K422R_FBP**^ **with PKM2 wild type bound with FBP**. (A) Close-up views of PKM2 mutants with critical residues indicated as stick representation. PKM2^Phe^ (in T-state) is colored in gray. (B) Close-up views of PKM2^K422R_FBP^ with critical residues indicated as stick representation. PKM2^FBP^ (in R-state, PDB: 4B2D) is colored in gray

The PKM2^K422R^ tetramer is structurally similar to the PKM2^Phe^ tetramer (PDB: 4FXJ) with a rmsd of 0.41 Å for 1720 aligned Cα atoms (Fig. 4A). Compared to PKM2^Phe^, PKM2^K422R^ has two additional hydrogen bonds between residue K422R on α15 on monomer C and E418 on α15 on monomer D, which may lead to the dissociation of helices α14 on monomer C and helices α15 on monomer D, and therefore strengthen intermolecular interactions (α15 on monomer C and α15 on monomer D) on the C-C′ interface to facilitate the T-state tetramer formation. The strong intermolecular interactions on the C-C′ interface in PKM2^K422R^ may be difficult to disrupt by PEP to form an R-state conformation. The above structural analyses support the observations that PKM2^K422R^ forms a less active tetramer in solution (compared to PKM2^WT^) but is stable in the thermal-shift assay (Figs. 1C, 1D and S1E).

Although PKM2^K422R^ tends not to adopt a PEP-induced R-state conformation because of strong intermolecular interactions on the C-C′ interface (compared to PKM2^Oxalate^), association of FBP did favor the R-state tetramer formation. Our structure of PKM2^K422R_FBP^ offers direct evidence for the R-state formation: the PKM2^K422R^ tetramer adopts a similar fold to that of PKM2^WT^ bound to FBP (PDB: 4B2D) (Fig. 4B). In support of the above observations, PKM2^K422R^ showed pyruvate kinase activity comparable to that of PKM2^WT^ in the presence of FBP, but significantly decreased activity and enhanced cooperativity in the absence of FBP (Fig. 1C and Table S1).

### PKM2^R399E^ tends to inhibit the formation of R-state tetramer

PKM2^R399E^ also adopts the T-state conformation (Fig. S2A). No significant difference existed between the structures of PKM2^R399E^ and PKM2^Phe^ (Fig. 4A). Residue R399 of α14 on monomer C forms hydrogen bonds with residues E396 of α14 and E418 of α15 on monomer D on the C-C′ interface of the PKM2^WT^ structure. However, residue R399E on monomer C may not form the same hydrogen bonds in PKM2^R399E^ even in the R-state (in the presence of PEP or FBP) because the three negatively charged residues may repel one another (Fig. 3). The existence of R399E in PKM2 will inhibit the formation of the R-state conformation induced by FBP or PEP, and lead to a decrease of enzymatic activity (compared to PKM2^WT^). Consistently, in the presence of FBP, PKM2^R399E^ is less active and has less of a tendency to form a tetramer compared to PKM2^WT^ (Fig. 1C and 1E). In contrast, no such significant difference in the extent of tetramer formation was observed in the absence of FBP.

### PKM2^Y105E^ inhibits FBP association and formation of the FBP-induced R-state of PKM2

A previous study indicated that phosphorylation of residue Y105 inhibits FBP association and tetramer formation of PKM2, and thus decreases its enzymatic activity (Hitosugi et al., 2009). In the crystal structure, PKM2^Y105E^ binds to proline, which was derived from a crystallization condition and might have facilitated a T-state conformation (as did phenylalanine) (Fig. S2D). No significant conformational change around residue Y105E was observed between structures of PKM2^Y105E^ and PKM2^Oxalate^ (Fig. 3). The crystal structure could not reveal the mechanism for the inhibition of FBP association. However, it is possible that phosphorylation of Y105 functions during the dynamic transition, which could not be observed in crystal structure. In support of this conjecture, PKM2^Y105E^ has similar enzymatic activity and tetramer formation (compared to PKM2^WT^) in the absence of FBP (Fig. 1C and 1D). The addition of oxalate leads to an increase in tetramer formation to a comparable extent for PKM2^Y105E^ and PKM2^WT^ (Fig. S1F). In contrast, the addition of FBP results in significantly less of an increase in the extent of tetramer formation for PKM2^Y105E^ (compared to PKM2^WT^) (Fig. 1E). Taken together, the mutation Y105E, or phosphorylation of residue Y105, inhibits FBP association and prevents the FBP-induced R-state formation of PKM2.

## Discussion

### A working model for the dynamic regulation of PKM2

**Figure 5:**
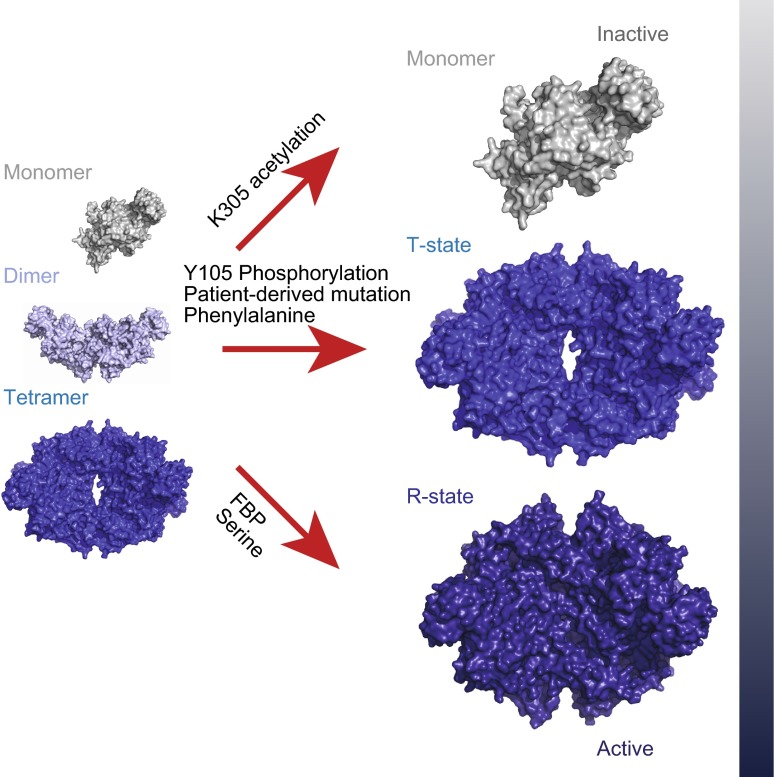
**Regulation of PKM2 activity by inhibitor/activators, post-translational modifications and a patient-derived mutation**. PKM2 exists in a mixed population of monomer, dimer and tetramer. Acetylation of residue K305 disrupts the tetramer formation. Phosphorylation of residue Y105, the patient derived mutation K422R and phenylalanine association induce an inactive T-state tetramer formation, whereas FBP and serine promote its active R-state formation. The level of pyruvate kinase activity of PKM2 is indicated by a colored bar, in which gray represents low activity and deep blue represents high activity

Based on above analyses, here we propose a working model for the dynamic regulation of PKM2 (Fig. 5). PKM2 exists in a mixed population of monomer, dimer and tetramer, and prefers a T-state conformation if the tetramer forms in the absence of FBP. Upon FBP association, PKM2 undergoes a change from the T-state to the R-state conformation, which favors the recognition of PEP in the active site and enhances its pyruvate kinase activity. Regulation of the intermolecular interactions on the A-A′ or C-C′ interface plays an important role in conformational changes of PKM2. A “Rock and Lock” model has been proposed based on structural studies of pyruvate kinase from *L. Mexicana* (Morgan et al., 2010). However, the model was not sufficient to reveal the mechanism for dynamic regulation of human PKM2. In this work, a “seesaw” model was proposed based on the comparison of human PKM2 structures in T- and R-state tetramers (Fig. 2B and 2C). Our biochemical and structural studies revealed the mechanisms for dynamic regulation of the pyruvate kinase activity of PKM2 by inhibitor/activator, post-translational modifications and a patient-derived mutation (Fig. 5). The two layers of regulation provide precise control of pyruvate kinase activity of PKM2 in various biological and pathological conditions.

Post-translational modifications (such as acetylation and phosphorylation) and the observed patient-derived mutation regulate PKM2 pyruvate kinase activity through modulating tetramer formation or the transition between T- and R-state conformations (Fig. 5). PKM2 with an acetylated K305 mainly forms a monomer in solution and forms a dimer in the presence of FBP because the protein loses the intermolecular interactions on the A-A′ interface. The lack of tetramer formation leads to a significant decrease in the enzymatic activity. Phosphorylation of residue Y105 inhibits FBP association and prevents the FBP-induced R-state formation of PKM2, but experiences minor effects in the absence of FBP. Acetylation of residue K433 was reported to decrease PKM2 activity through inhibiting FBP association (Lv et al., 2013). PKM2^R399E^ tends to inhibit the formation of R-state tetramer (R399E on monomer A repels E396 on monomer A and E418 on monomer B on the C-C′ interface) and leads to a decrease of activity even in the presence of FBP. Interestingly, PKM2^K422R^ tends not to form the R-state conformation in the absence of FBP because the hydrogen bonds between residues K422R on monomer A and E418 on monomer B on the C-C’ interface favor a stable T-state tetramer formation. Moreover, FBP could induce the re-formation of the R-state tetramer of PKM2^K422R^. Thus, PKM2^K422R^ shows low activity and high cooperativity in the absence of FBP but comparable activity to that of PKM2^WT^ in the presence of FBP. Notably, the cellular concentration of PEP ranges from 4 μmol/L to 140 μmol/L in human and rat tissues (Liebermeister, 2005). Under such conditions, PKM2^K305Q^ and PKM2^K422R^ show a significant decrease in pyruvate kinase activity (compared to PKM2^WT^). Consistently, acetylation of K305 on PKM2 changes metabolic intermediates and promotes cell proliferation and tumor growth, and the K422R mutation of PKM2 enhances HeLa cell proliferation (Gupta et al., 2010; Lv et al., 2011).

The pyruvate kinase activity of PKM2 could be regulated by small molecules. FBP could recruit the FAM to the FBP binding pocket on the C-C′ interface and facilitate R-state tetramer formation of PKM2. PEP, or its analog (such as oxalate, malonate), associates with PKM2 on the A-A′ interface and allosterically induces R-state tetramer formation. Another PKM2 activator, TEPP-46, enhances the intermolecular interactions on the A-A′ interface and favors an R-state conformation (Anastasiou et al., 2012). The crystal structure of PKM2 bound to phenylalanine demonstrates that the amino acid could lock PKM2 in an inactive T-state (Morgan et al., 2013), while the crystal structure of PKM2 bound to serine demonstrates that the amino acid could also allosterically activate PKM2 (Chaneton et al., 2012). SAICAR is a metabolite abundant in proliferating cells and stimulates both pyruvate and protein kinase activities of PKM2 (Keller et al., 2012; Keller et al., 2014). PKM2^Q393K^ (Q393 is located on helix α14) is a SAICAR-insensitive mutant, suggesting that the compound might regulate PKM2 through both A-A′ and C-C′ interfaces.

PKM2 has long been known to have pyruvate kinase activity. However, several studies recently indicated that PKM2 also possesses protein kinase activity, which plays an important role in tumorigenesis. For example, EGFR-activated ERK2 promotes nuclear translocation and protein kinase activity of PKM2 (Yang et al., 2012a; Yang et al., 2012b). SAICAR directly interacts with PKM2 and promotes its protein kinase activity (Keller et al., 2014). In addition, association of tyrosine phosphorylated peptide, acetylation of residue K433 or the mutation of R399E of PKM2 promotes its protein kinase activity and nuclear localization, which are correlated with predominant dimer formation (Gao et al., 2012; Gao et al., 2013; Lv et al., 2013). Interestingly, these dimers are formed with the loss of intermolecular interaction on the C-C′ interface, suggesting that regulation on this interface may be responsible for the switch from pyruvate kinase activity to protein kinase activity of PKM2. Further structural and biochemical studies may reveal the mechanisms for the switch of substrate specificity of PKM2.

In summary, our study provides structural insight into dynamic regulation of PKM2 by post-translational modifications and a patient-derived mutation. The “seesaw” model reveals the mechanism for the transition between T- and R-state conformations and provides a platform for further investigation of other modifications/mutations of PKM2.

## Accession numbers

The atomic coordinates of the PKM2^Y105E^, PKM2^K305Q^, PKM2^R399E^, PKM2^K422R^, and PKM2^K422R_FBP^ have been deposited in the Protein Data Bank with the PDB codes 4QG6, 4QG8, 4QG9, 4QGC, and 4RPP, respectively.

## Materials and methods

### Protein crystallization

The ORF of human PKM2 was kindly gifted from Kun-liang Guan’s lab at Fudan University. Procedures for mutagenesis and protein purification can be found in the Supplemental Materials. The crystals for PKM2 mutants were obtained at 18°C by the hanging-drop, vapor-diffusion method by mixing 1 μL protein solution (15–20 mg/mL) with 1 μL reservoir solution containing 0.2 mol/L malonate pH 5.0, 20% PEG3350 (for PKM2^K305Q^); 0.2 mol/L L-proline, 0.1 mol/L HEPES pH 7.5, 24% PEG1500 (for PKM2^Y105E^); 0.2 mol/L CaAc_2_, 20% PEG3350 (for PKM2^R399E^); 0.2 mol/L K_2_SO_4_, 20% PEG3350 (for PKM2^K422R^); and 0.1 mol/L sodium chloride, 0.1 mol/L BIS-TRIS propane pH 9.0, 25% PEG 1500 for (PKM2^K422R_FBP^).

### Data collection and structure determination

Crystals were mounted on nylon loops and flash-cooled in a cold nitrogen stream at 100 K. The data sets were collected at a single wavelength at the Shanghai Synchrotron Radiation Facility (SSRF) in China on beamline BL17U. Data were indexed, integrated and scaled using the HKL2000 program (Otwinowski and Minor 1997). The structures of PKM2 mutants were determined by molecular replacement using the monomer of PKM2 wild-type structure (3BJT.pdb) as a searching model (Christofk et al., 2008b). Details about structure determination and refinement can be found in Supplemental Materials.

### Measurement of pyruvate kinase activity

A lactate dehydrogenase (LDH)-coupled pyruvate kinase activity assay was performed to measure the activity of wild-type PKM2 and mutants of PKM2 as previously described (Morgan et al., 2013). Because the reaction was sigmoidal, the reaction rates of PKM2 (in the absence of FBP) were calculated according to the Allosteric Sigmoidal Equation. For the reaction rates of PKM2 in the presence of FBP, we performed the calculation according to Michaelis-Menten Equation. Details for the pyruvate kinase assay can be found in Supplemental Materials.

### Thermal shift assay and gel filtration assay

The thermal-shift assay and the gel-filtration (Superdex 200, GE healthcare, 10/300 GL) assay were performed to examine the thermal stability and tetramer formation of PKM2 proteins. Detailed descriptions can be found in Supplemental Materials.

## Electronic supplementary material

Below is the link to the electronic supplementary material.

Supplementary material 1 (PDF 1208 kb)

## Abbreviations

ADP: adenosine di-phosphate
ATP: adenosine tri-phosphate
FBP: fructose 1,6-bisphosphate
PEP: phosphoenolpyruvate
PKM2: pyruvate kinase isoform M2
