# Supplementary material for: Structural insight into mechanisms for dynamic regulation of PKM2
Source: Protein Cell. 2015 Feb 4;6(4):275–87. doi: 10.1007/s13238-015-0132-x (PMC4383751; doi:10.1007/s13238-015-0132-x)
Supplement: Supplementary file 1 — Supplementary material 1 (PDF 1208 kb) [file 13238_2015_132_MOESM1_ESM.pdf]

Supplemental Materials

Supplemental Figures

Supplemental Figure 1. Purification, gel-filtration and thermal shift assay of PKM2 proteins

Supplemental Figure 2. Structures of wild-type and mutants of PKM2

Supplemental Figure 3. Sequence and secondary structure of human PKM2

Supplemental Tables

Supplemental Table1: Kinetic activities and thermal stability of wild-type and mutants of PKM2

Supplemental Table 2: Distances between representative residues for “see-saw” model

Supplemental Experimental Procedures

Supplemental References

Supplemental Figures

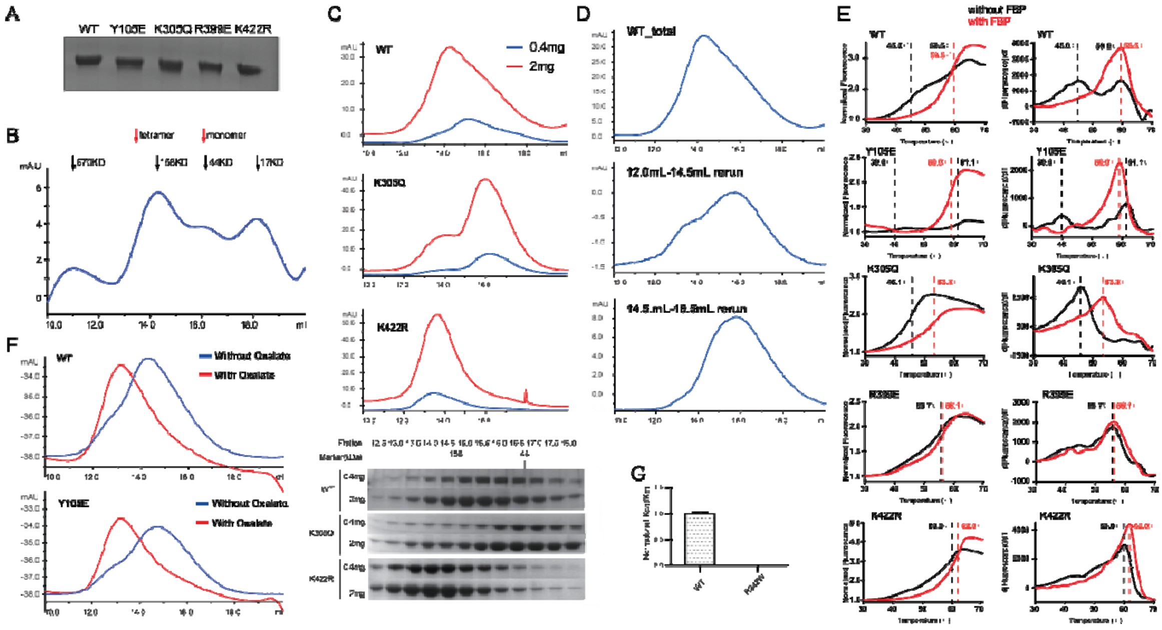

Supplemental Figure 1. Purification, gel-filtration and thermal shift assay of PKM2 proteins

(A) Protein purification of wild-type and mutants of PKM2. Equal amount of PKM2 proteins were subjected to SDS-PAGE and stained by Coomassie blue.

(B) Standards for gel-filtration with molecular weight and peak positions for PKM2monomer and tetramer indicated above. Gel-filtration was performed using Superdex 200 (GE healthcare, 10/300 GL).

(C) Gel filtration for wild-type and mutants of PKM2 in two different protein concentrations (0.4 mg/mL and 2.0 mg/mL). The samples were subjected to SDS-PAGE and Comassie blue staining. Gel-filtration was performed using Superdex 200 (GE healthcare, 10/300 GL).

(D)Gel filtration for wild-type PKM2. The monomeric and tetrameric samples were re-subjected for gel filtration as described in (C).

(E) Representative thermal shift profiles for PKM2 proteins in the absence (black line) or presence (red line) of FBP (500 μM).

(F) Gel filtration for $PKM2^{WT}$and $PKM2^{Y105E}$ in the presence or absence of oxalate.

(G) Normalized activities (kcat/Km) of wild-type and mutation R342W of PKM2 with the value (kcat/Km) of $PKM2^{WT}$ as a standard. The error bars represent mean± SD for triplicate experiments.

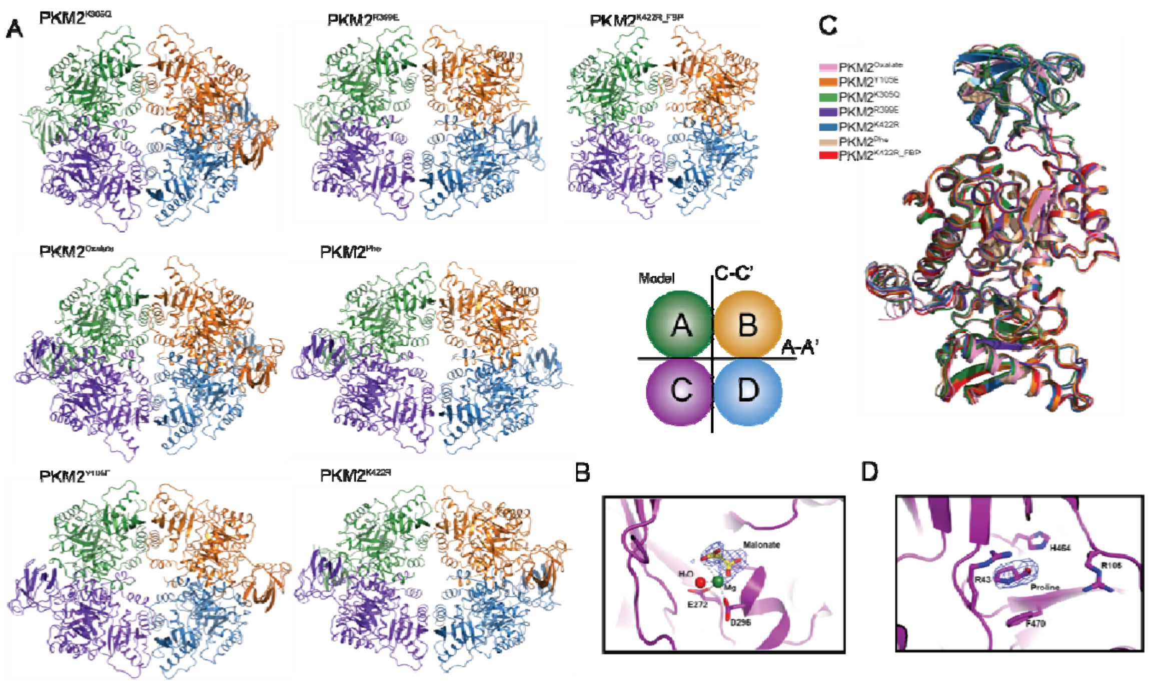

Supplemental Figure 2. Structures of wild-type and mutants of PKM2

(A) Ribbon representation of PKM2 proteins with each monomer indicated in different color. Note that PKM2^{Oxalate} is PKM2^{WT} bound to oxalate, and PKM2^{Phe} is PKM2^{R489A}bound to Phenylalanine.

(B) Ribbon representation of PKM2^{K305Q} Structure in close-up view. Malonate is located in the active site and favors the formation of R-state tetramer. Malonate is shown in stick representation with corresponding electron density map (2Fo-Fc) contoured at 1.0 σ and shown in blue mesh.

(C) Structural comparison of monomers from each PKM2 structure with the color scheme indicated.

(D) Ribbon representation of PKM2^{Y105E}Structure in close-up view. Proline is located in the allosteric effector binding site. Proline is shown in stick representation with corresponding electron density map (2Fo-Fc) contoured at 1.0 σ and shown in blue mesh.

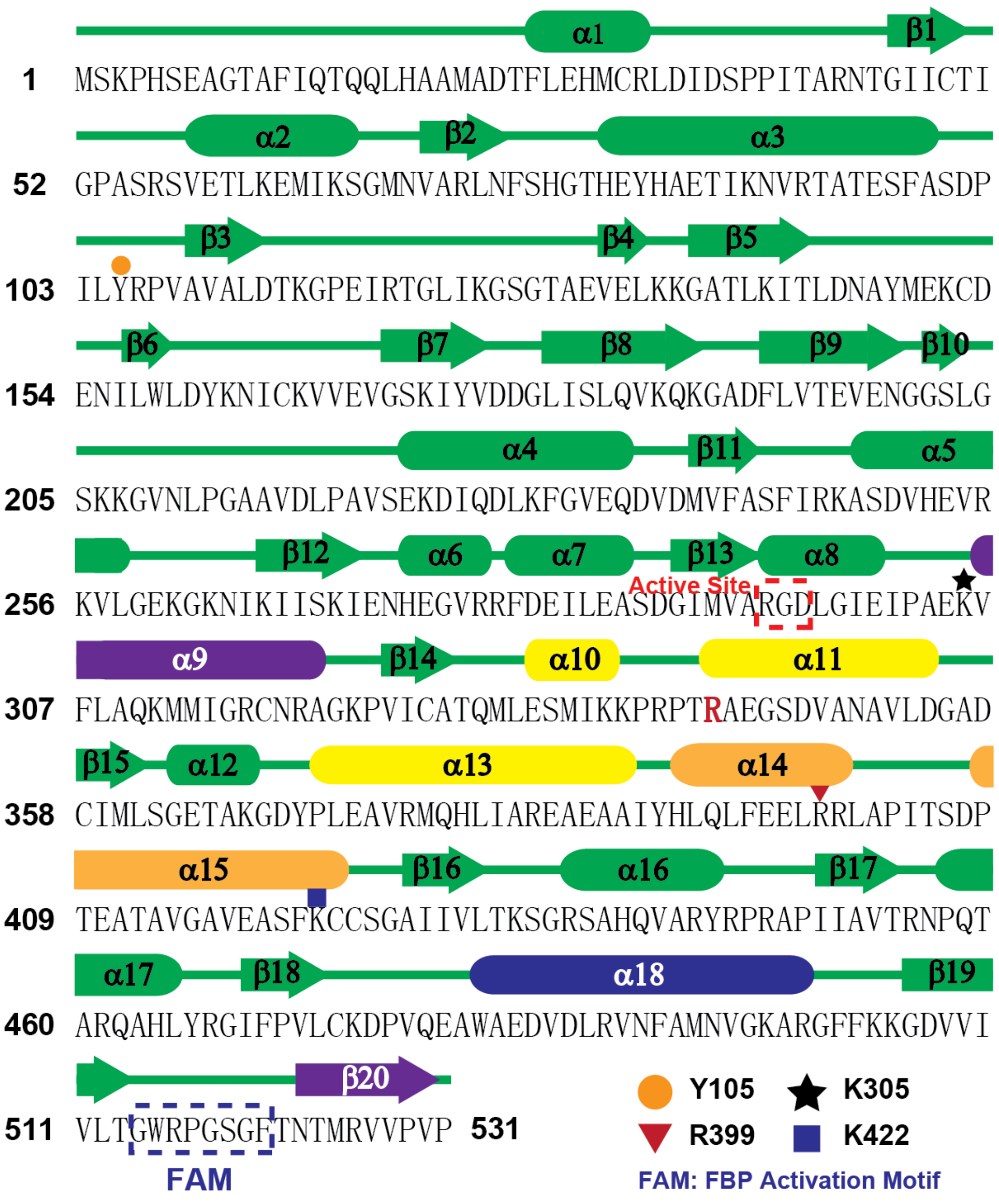

Supplemental Figure 3. Sequence and secondary structure of human PKM2

The secondary structure of PKM2 is indicated above the sequence. Critical residues and secondary structures of PKM2 studied in this work are indicated. Helixα9and strand β20 are the axis for “see-saw” model and highlighted in purple. The residues at the active site are highlighted by red box. Residue R342, which is responsible for active site stabilization, is colored in red. FAM and α18 involved in the C-C’ interface are

highlighted in blue, and α10/ α11/ α13involved in the A-A' interface are highlighted in yellow. Helices α14 and α15 in the A-A' and C-C' interfaces are highlighted in orange.

Supplemental Tables

Supplemental Table1: Kinetic activities and thermal stability of wild-type and mutants of PKM2

Protein	V (Units / min)	Km (mM)	nH	Vmax/Km ((mol/L)$^{-1}$s^{-1})	Normalized kcat/Km	Tm (℃)
WT	135.0 (11.8) [a]	0.6 (0.1)	1.6 (0.2)	228.3 (15.3)	1 (0.1)	44.2 (0.8) / 60.4 (0.6)
Y105E	92.8 (3.0)	0.3 (0.0)	1.5 (0.1)	267.8 (2.9)	1.2 (0.0)	40.5 (0.5) / 59.7 (1.0)
K305Q	ND[b]	ND	ND	ND	ND	43.5 (1.6)
R399E	117.9 (2.7)	0.9 (0.0)	2.2 (0.2)	133.6 (7.1)	0.6 (0.0)	55.8 (0.1)
K422R	79.6 (1.3)	2.2 (0.0)	2.1 (0.1)	36.2 (0.8)	0.2 (0.0)	60.8 (0.5)
WT (FBP)	311.3 (5.6)	0.2 (0.0)	1.0	1282.5 (71.7)	5.6 (0.3)	59.1 (0.5)
Y105E (FBP)	180.9 (3.1)	0.2 (0.0)	1.0	975.4 (102.1)	4.3 (0.4)	58.4 (0.5)
K305Q (FBP)	36.4 (1.7)	0.6 (0.1)	1.0	58.4 (2.8)	0.3 (0.0)	53.8 (0.4)
R399E (FBP)	204.4 (4.5)	0.5 (0.0)	1.0	418.8 (18.9)	1.8 (0.1)	56.4 (0.2)
K422R (FBP)	316.6 (5.8)	0.4(0.0)	1.0	881.1 (52.3)	3.9 (0.2)	61.3 (0.3)

[a]: The value in parentheses indicates standard deviation and the experiments are repeated three times.

[b]: Not Determined.

Supplemental Table2: Distances between representative residues for “see-saw” model

Protein	State	Distance between residues on C-C’ interface (Å)				Distance between residues on A-A’ interface (Å)			
		E397 (α14)	M525 (β20)	L488 (α18)	A-β20 C-β20	E373 (α13)	K311 (α9)	V414 (α15)	A-α9 C-α9
$PKM2^{Oxalate}$	R	14.2	5.0	27.0	59.6	31.2	18.0	40.3	43.7
$PKM2^{K305Q}$	R	13.8	5.0	27.8	59.6	31.1	17.8	40.7	43.0
$PKM2^{K422R\_FBP}$	R	16.2	4.9	27.1	57.5	33.3	18.2	40.0	47.7
$PKM2^{Y105E}$	T	15.9	4.8	25.9	56.5	38.0	18.0	36.5	50.4
$PKM2^{R399E}$	T	20.7	5.1	25.7	53.9	37.7	18.2	36.4	51.6
$PKM2^{K422R}$	T	20.6	5.0	25.9	53.6	37.8	18.1	36.2	52.1
$PKM2^{Phe}$	T	20.8	5.0	25.9	53.6	37.6	18.0	36.1	51.9

Note: All distances were measured between the two Cα atoms of the residues.

Supplemental Experimental Procedures

Protein Expression and Purification

The human full length PKM2 (1-531) was subcloned into modified pGEX-6P-1 vector. Mutations of PKM2 were generated by the Quick-Change mutagenesis protocols (Stratagene). All plasmids were verified by DNA sequencing and transformed into *Escherichia coli* strain BL21(DE3). Target proteins were overexpressed in 2xYT medium at 20°C overnight and induced by adding isopropyl-β-D-thiogalactopyranoside to 1mM. Cell expressing PKM2 were harvested and lysed in the buffer containing 20mMTris (pH 8.0), 200mMNaCl, 100mMKCl, 10mM MgCl2 supplemented with protease inhibitors and DNase. The supernatant were load to Ni-NTA affinity column (GE healthcare) and the fusion proteins on beads were digested with PreScission protease overnight at 4°C. Then the eluted proteins were further purified by gel filtration chromatography on a Superdex 200 (GE Healthcare, 10/300 GL). The peak fractions were collected and concentrated to 15-20mg/ml for crystallization.

Structure Determination and Refinement

The structures of PKM2 mutants were determined by molecular replacement using the monomer from wild-type PKM2 structure (3BJT.pdb) as a searching model(Christofk et al., 2008). The Matthews coefficients were calculated to estimate the number of molecules in one asymmetry unit. Rotation and translation search were performed using PHASER(Winn et al., 2011). All biased model were removed and rebuilt manually with COOT(Emsley and Cowtan, 2004). All refinements were performed using the refinement module phenix.refine of PHENIX package(Adams et al., 2002). Brief structure refinement procedure was as following. Rigid body refinement was conducted after initial

molecular replacement. After several cycles of manual building in Coot, XYZ coordinate, individual B factor, TLS, NCS restraints and weight optimization refinement were performed. The model quality was checked with the PROCHECK program(Laskowski et al., 1993), which shows a good stereochemistry according to the Ramachandran plot. PYMOL was used to generate the structure figures(DeLano, 2002).

Pyruvate Kinase Assay

Lactate dehydrogenase (LDH) coupled pyruvate kinase activity assay was carried out as described previously(Morgan et al., 2013). In brief, the reactions were performed at 37°C using PEP titration to calculate the kinetic parameters. PEP concentration spans from 39μM to 5mM. The reaction buffer system is PBS-CM pH7.4 buffer supplemented with 4 mM ADP, excessive amount of purified LDHA (homo-made), 20 mM MgCl2, 200 mM KCl, 1 mM DTT, and 1 mM NADH. All original data in the “Without FBP” reactions were fit to the allosteric sigmoidal equation using GraphPad Prism 5. The data in the “With FBP” reactions with 500μM FBP were fit to the Michaelis-Menten equation. Three independent experiments were performed and errors are shown in parentheses.

Allosteric Sigmoidal Equation: Y=Vmax*X^h/(Kprime + X^h). Vmax is the maximum enzyme velocity with the same units as Y. Kprime is related to Km and computed as Km^h. Km is the concentration of substrate that produces a half-maximal enzyme velocity .h is the Hill slope. When h=1, this equation is identical to the standard Michaelis-Menten equation.

Michaelis-Menten Equation: Y = Vmax*X/(Km + X). Vmax is the maximum enzyme velocity with the same units as Y. Km is the Michaelis-Menten constant, with the same units as X.

Thermal Shift Assay

Solutions containing 5 μL of 0.8 mg/mL protein, 4μL of 10x TSA buffer (200mM HEPES pH 7.4, 50 mM MgCl2, 500 mM KCl, 1M NaCl), 1 μL of SYPRO Orange (diluted 1/50 in 20mM HEPES pH7.4, purchased from Sigma) were added to the Strips 8 PCR tubes and the final volume were adjust to 40μL. With the presence of FBP, its concentration is 500 μM. The Strips 8 PCR tubes were heated in an i-Cycler iQ5 real-time PCR detection system (Bio-Rad) from 15°C to 85°C with an increment of 1°C. The Fluorescence changes for each tube were detected by a charge-coupled (CCD) camera. The melt temperature for the protein unfolding transition was analysis by TSA software(Life Technology). The final curve was fitted by GraphPad Prism 5.

Gel filtration assay

Equal amount of purified wild-type and mutants ofPKM2 were loaded onto a Superdex 200 column (GE healthcare, 10/300 GL) using 1xPBS buffer pH 7.4. Samples with FBP or with oxalate were incubated for one hour on ice before loading. The running buffer for the reactions containing FBP or oxalate included 500 μM FBP and 100 μM oxalate. Protein peak wad detected by 280nm absorbance.

Compliance with Ethics Guidelines

Ping Wang, Chang Sun, Tingting Zhu and Yanhui Xu declare that they have no conflict of interest.

This article does not contain any studies with human or animal subjects performed by the any of the authors.

Supplemental References

Adams, P.D., Grosse-Kunstleve, R.W., Hung, L.W., Ioerger, T.R., McCoy, A.J., Moriarty, N.W., Read, R.J., Sacchettini, J.C., Sauter, N.K., and Terwilliger, T.C. (2002). PHENIX: building new software for automated crystallographic structure determination. Acta Crystallogr D Biol Crystallogr *58*, 1948-1954.

Christofk, H.R., Vander Heiden, M.G., Wu, N., Asara, J.M., and Cantley, L.C. (2008). Pyruvate kinase M2 is a phosphotyrosine-binding protein. Nature *452*, 181-186.

DeLano, W.L. (2002). The PyMOL Molecular Graphics System. http://www.pymol.org.

Emsley, P., and Cowtan, K. (2004). Coot: model-building tools for molecular graphics. Acta Crystallogr D Biol Crystallogr *60*, 2126-2132.

Laskowski, R.A., MacArthur, M.W., Moss, D.S., and Thornton, J.M. (1993). PROCHECK: a program to check the stereochmical quality of protein structures. J. Appl. Cryst *26*, 283-291.

Morgan, H.P., O'Reilly, F.J., Wear, M.A., O'Neill, J.R., Fothergill-Gilmore, L.A., Hupp, T., and Walkinshaw, M.D. (2013). M2 pyruvate kinase provides a mechanism for nutrient sensing and regulation of cell proliferation. Proc Natl Acad Sci U S A *110*, 5881-5886.

Winn, M.D., Ballard, C.C., Cowtan, K.D., Dodson, E.J., Emsley, P., Evans, P.R., Keegan, R.M., Krissinel, E.B., Leslie, A.G., McCoy, A., *et al.* (2011). Overview of the CCP4 suite and current developments. Acta Crystallogr D Biol Crystallogr *67*, 235-242.
